# Post-Transcatheter Aortic Valve Replacement Infective Endocarditis Leading to Mitral Anterior Leaflet Perforation: A Case Report

**DOI:** 10.7759/cureus.35049

**Published:** 2023-02-16

**Authors:** Amman Yousaf, Muhammad Ahmad, Khadija Qureshi, Hafiz Khan, Ahmad Munir

**Affiliations:** 1 Internal Medicine, McLaren Flint, Flint, USA; 2 Cardiology, Mclaren Flint, Flint, USA; 3 Cardiology, McLaren Flint, Flint, USA

**Keywords:** heart failure, group b streptococcus, mitral valve perforation, transcatheter aortic valve replacement, infective endocarditis

## Abstract

Transcatheter aortic valve replacement(TAVR)-related infective endocarditis is a rare but fatal complication that can lead to mitral valve perforation. The clinical presentation usually includes rapidly progressive heart failure and mitral regurgitation. Transesophageal echocardiogram (TEE) is considered superior to transthoracic echocardiogram (TTE) in delineating the diagnosis of mitral valve perforation. We present a case of a 75-year-old female who had a TAVR for severe aortic stenosis three years ago and presented with new-onset atrial fibrillation and developed rapidly progressive acute decompensated heart failure. A TTE showed echogenic vegetation of the mitral valve with a perforated mitral anterior leaflet and mitral regurgitation. The blood cultures grew Group B Streptococcus, and our patient lacked the risk factors for infective endocarditis, including alcoholism, chronic liver disease, pregnancy, immunosuppression, or malignancy. This article highlights infective endocarditis with an uncommon pathogen in a patient with a prior TAVR that leads to the fatal complication of mitral valve perforation.

## Introduction

Mitral valve perforation is an infrequent complication of infective endocarditis, which can be fatal if not managed timely. Infective endocarditis is highly prevalent in intravenous drug users, immunodeficient patients, patients with HIV/AIDS, and patients with abnormal or prosthetic heart valves [1]. Mitral valve perforation and aneurysms of the mitral valve are commonly caused by infective endocarditis. Mitral valve perforation can result in a sudden mitral regurgitation with a decrease in the ejection fraction, and patients can present with dyspnea, acute pulmonary edema, chest pain, and cardiogenic shock [2,3]. We present the case of a 75-year-old female who had a transcatheter aortic valve replacement (TAVR) and presented with sudden heart failure and was found to have mitral valve infective endocarditis (IE) with anterior leaflet perforation and severe mitral regurgitation.

## Case presentation

A 75-year-old Caucasian female with a medical history of coronary artery disease status post stenting in the proximal left anterior descending artery, severe aortic stenosis status post-TAVR (three years before this admission, right transfemoral valve replacement using 23 mm SAPIEN S3 TAVR valve), type 2 diabetes mellitus, hyperlipidemia, and a 10-pack-a-year practice of smoking presented to the hospital after a mechanical fall at home. She did not lose consciousness, and her trauma workup was negative for any fractures or intracranial bleeding. Her presenting vitals were as follows: heart rate of 79 beats per minute, respiratory rate of 17 breaths per minute, blood pressure of 137/83 mmHg, a temperature of 37.2 degree Celsius, and SpO2 of 94% in room air. Her complete blood count and metabolic profile were within normal limits.

**Figure 1:**
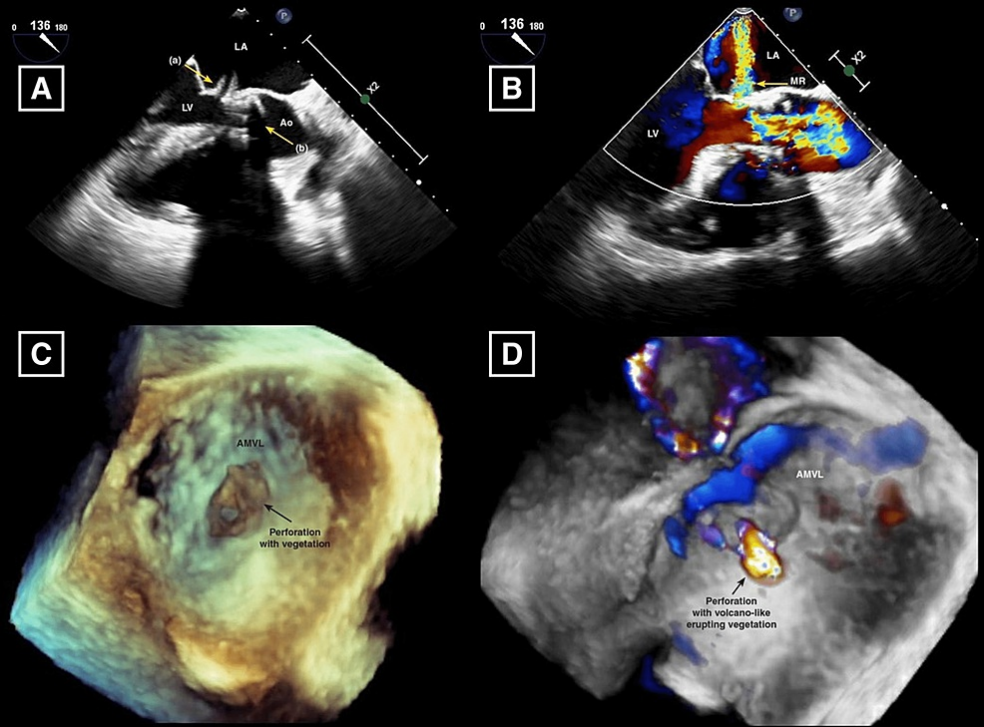
Transesophageal echocardiogram. A: 2D three-chamber view showing anterior mitral leaflet valve perforation with vegetation (yellow arrow A) along the TAVR (yellow arrow B). B: Doppler three-chamber view showing moderate to severe mitral regurgitation jet through the perforation. C: 3D view demonstrates anterior mitral leaflet valve (AMLV) perforation with attached vegetation. D: Doppler 3D view showing volcano-like vegetation coming out of AMLV perforation.

A few hours after hospitalization, the patient developed a new-onset atrial fibrillation with a rapid ventricular response. The patient was started on intravenous amiodarone and heparin drip (CHA2DS2VASc: 5). On day two of hospitalization, the patient developed shortness of breath, fever of 39.1 degree Celsius, and hypotension with mean arterial pressure of 48 mmHg. She was resuscitated with fluids and was started on vasopressors, and her respiratory status deteriorated, warranting intubation and ventilatory support. Chest X-ray showed pulmonary congestion. Repeat labs revealed leukocytosis of 21 X 109/L and elevated lactic acid of 9.1 mg/dL. The transthoracic echocardiogram echogenic vegetation on the mitral valve and severe mitral regurgitation. For further evaluation, a transesophageal echocardiogram was performed, which showed a left ventricular ejection fraction of 40%, severe pulmonary hypertension with RVSP of 106 mmHg, and perforation of the anterior portion of the mitral valve annulus that resulted in severe mitral regurgitation. There was echogenic vegetation along the anterior portion of the mitral valve annulus; however, there was no vegetation on the prosthetic valves (Figure 1).

Her blood cultures grew Group B Streptococcus (streptococcus agalactia), and she was started on antibiotics. Treatment options for open heart surgery and mitral valve repair were discussed. However, after a discussion with the patient's family, it was decided that the patient would undergo medical management. Nevertheless, her hemodynamic status did not improve, and she died on day three of hospitalization.

## Discussion

TAVR is usually well tolerated; however, it can result in a range of early periprocedural, device-related, and late complications [3]. Leon et al. [4] specified various TAVR-related complications, such as stroke, myocardial infarction, bleeding, acute kidney injury, vascular complications, prosthetic valve-related complications, endocarditis, arrhythmias, and coronary obstruction. We reported the case of a patient who developed heart failure because of mitral valve anterior leaflet perforation and infective endocarditis three years after TAVR. Mitral valve perforation is a rare but well-known complication of TAVR, especially in cases of the low deployed valve. The mechanical abrasions from the contact of the prosthetic aortic valve with the anterior mitral leaflet can predispose the mitral valve to infective endocarditis and perforation [5]. It is still unclear in the literature whether endocarditis precedes mitral valve perforation or vice versa; nevertheless, Miura et al. [6] described a case of anterior mitral leaflet perforation without infective endocarditis.

The incidence of early and late infective endocarditis is almost equal among surgical aortic valve replacement and TAVR [5]. Based on the onset after the valve replacement, prosthetic valve endocarditis can be classified into early (within two months), intermediate (2 to 12 months), and late (>12 months) [7]. The prevalence of infective endocarditis has been demonstrated between 0 to 2.3% during 1 to 3 years after the procedure. Eisen et al. demonstrated underlying organisms for infective endocarditis in TAVR patients i.e., staphylococcus, streptococcus, and atypical organisms such as Enterococcus faecium, Corynebacterium, Moraxella, Histoplasma, and Candida species [8]. The incidence of infective endocarditis secondary to Group B Streptococci (GBS) infection is rare, with GBS accounting for merely 1.7% of cases. However, there are no reported cases of Group B streptococcus infective endocarditis in a TAVR patient in the literature. Although there are no separate criteria for diagnosing infective endocarditis in TAVR patients, Duke criteria are still considered the gold standard.

The incidence of infective endocarditis secondary to Group B streptococci (GBS) infection is rare, with GBS accounting for merely 1.7% of cases. The risk factors for developing GBS-associated sepsis and infective endocarditis include alcoholism, hepatic cirrhosis, immunosuppression, diabetes, pregnancy, and malignancy [9]. Our patient lacked any of these risk factors. There are several reported cases of TAVR infective endocarditis in the literature, which resulted in mitral valve leaflet perforation or rupture and was associated with increased mortality [5,10-13]. Most cases were diagnosed on transthoracic or transesophageal echocardiogram, and TEE was considered superior in detecting mitral valve perforation or rupture. The literature still lacks any specific management guidelines for mitral valve perforation related to TAVR, and management decisions are made case by case.

## Conclusions

TAVR can result in abrasion of the mitral valve, increasing the risk of infective endocarditis. TAVR-related late infective endocarditis leading to mitral valve perforation is a relatively rare complication that can be fatal without surgical intervention. It needs a high degree of suspicion, especially in patients lacking other risk factors.
